# Status of insecticide susceptibility in *Anopheles arabiensis* and detection of the knockdown resistance mutation (kdr) concerning agricultural practices from Northern Sudan state, Sudan

**DOI:** 10.1186/s43141-021-00142-1

**Published:** 2021-03-29

**Authors:** M. Y. Korti, T. B. Ageep, A. I. Adam, K. B. Shitta, A. A. Hassan, A. A. Algadam, R. M. Baleela, H. A. Saad, S. A. Abuelmaali

**Affiliations:** 1 Tropical Medicine Research Institute, National Center for Research, Khartoum, Sudan; 2 Department of Biological Sciences, Federal University Lokoja, Lokoja, Kogi State Nigeria; 3 Department of Zoology, Faculty of Sciences, University of Khartoum, Khartoum, Sudan; 4 Department of Medical Entomology, National Public Health Laboratory, Federal Ministry of Health, Khartoum, Sudan

**Keywords:** *Anopheles arabiensis*, Insecticide resistance, *kdr*, KDT50, KDT95

## Abstract

**Background:**

Chemical control has been the most efficient method in mosquito control, the development of insecticide resistance in target populations has a significant impact on vector control. The use of agricultural pesticides may have a profound impact on the development of resistance in the field populations of malaria vectors. Our study focused on insecticide resistance and knockdown resistance (*kdr*) of *Anopheles arabiensis* populations from Northern Sudan, related to agricultural pesticide usage.

**Results:**

*Anopheles arabiensis* from urban and rural localities (Merowe and Al-hamadab) were fully susceptible to bendiocarb 0.1% and permethrin 0.75% insecticides while resistant to DDT 4% and malathion 5%. The population of laboratory reference colony F189 from Dongola showed a mortality of 91% to DDT (4%) and fully susceptible to others. GLM analysis indicated that insecticides, sites, site type, and their interaction were determinant factors on mortality rates (*P* < 0.01). Except for malathion, mortality rates of all insecticides were not significant (*P* > 0.05) according to sites. Mortality rates of malathion and DDT were varied significantly (*P* < 0.0001 and *P* < 0.05 respectively) by site types, while mortality rates of bendiocarb and permethrin were not significant (*P* >0.05). The West African *kdr* mutation (L1014F) was found in urban and rural sites. Even though, the low-moderate frequency of *kdr* (L1014F) mutation was observed. The findings presented here for *An. arabiensis* showed no correlation between the resistant phenotype as ascertained by bioassay and the presence of the *kdr* mutation, with all individuals tested except the Merowe site which showed a moderate association with DDT (OR= 6 in allelic test), suggesting that *kdr* genotype would be a poor indicator of phenotypic resistance.

**Conclusion:**

The results provide critical pieces of information regarding the insecticide susceptibility status of *An. arabiensis* in northern Sudan. The usage of the same pesticides in agricultural areas seemed to affect the Anopheles susceptibility when they are exposed to those insecticides in the field. The kdr mutation might have a less role than normally expected in pyrethroids resistance; however, other resistance genes should be in focus. These pieces of information will help to improve the surveillance system and The implication of different vector control programs employing any of these insecticides either in the treatment of bed nets or for indoor residual spraying would achieve satisfactory success rates.

## Background

Malaria causes considerable morbidity and mortality in Sudan, especially among young children and pregnant women. In northern Sudan, 16% of hospital deaths are attributed to the disease. The case fatality rate of inpatient malaria cases is reported to be 2.5% [1].

*Anopheles arabiensis* Patton is a member of *An. gambiae* Gillies complex and the third most important malaria vector in Africa [2], while in Sudan this species is the principal malaria vector in most regions of the country, and the only known malaria vector mosquito in Northern and Central Sudan [3, 4].

The use of long-lasting insecticidal nets (LLIN) and indoor residual spraying (IRS) with WHO-approved insecticides are considered as two of the main strategies of preventing transmission of malaria and control in Sudan which is used on a large scale [5]. Pyrethroids are one of the most recommended classes of insecticide approved for LLINs because they are fast-acting and long-lasting and demonstrate relatively low toxicity to mammals. The control strategies, even though, have been hampered by the development and spread of vector resistance to insecticides, a growing problem in many African countries [5].

The use of insecticide has been the most successful way of controlling mosquitoes; the development of resistance in target populations has a significant impact on vector control, and ultimately on the prevalence of malaria. In recent year, insecticide resistance is a growing concern in many countries which requires immediate attention due to a pronounced increase in the use of insecticides for malaria control. These same insecticide classes are also widely used to control agricultural pests in Africa, and this has posed an additional selection pressure on mosquitoes when insecticide contaminated groundwater permeates their larval habitats. The intensive exposure to insecticides has resulted in the evolution of insecticide resistance in the *Anopheles* mosquito and other disease vectors [6].

The use of agricultural pesticides may have a weighty impact on the development of resistance in the field populations of malaria vectors. The practice of using pesticides was common in northern and central Sudan. Organophosphates and carbamates were the most commonly applied pesticides [7, 8].

Insecticide resistance to a range of insecticides in *An. arabiensis* has been reported in several different countries in Africa [9–12]. In Sudan malathion, DDT, dieldrin, bendiocarb, deltamethrin, and permethrin were reported [4, 7, 13–16].

The development and spread of malaria vector resistance to insecticides have been attributed to the intensive use of insecticides in agriculture, particularly in cotton cultivation [17, 18].

The DDT is a chloro which was banned since three decades ago, it is considered an organic persistent pollutant and its action affect the central nervous system, producing hyperactivity and tremor. Malation belongs to organophosphate class, is a rapidly metabolized and eliminated, and inhibits the acetyl-cholinesterase. Bendiocarb is a carbamate, relatively fast acting through inhibiting the cholinesterase activity which is rapidly revisable. Permethrin is a pyrethroid, neurotoxin, acting in the nervous system causing repetitive nerve action.

Pyrethroids and DDT target the voltage-gated sodium channel site. Two alternative substitutions of the leucine 1014 residue can lead to target site resistance. The 1014F allele was first identified in strains of *An. gambiae* from Burkina Faso and Côte d’Ivoire [19] and the 1014S allele was later identified in this species in Kenya [20].

In *An. arabiensis*, L014F allele has been found in several widely dispersed populations from Burkina Faso [21], Tanzania [22], Sudan [4, 15], Senegal [23], and Ethiopia [11, 12]. The L014S allele was also observed in wild populations of *An. arabiensis* from Uganda [24] and western Kenya [25]. Both L014F and 1014S alleles have been detected together in the same populations in Sudan [15] and Cameroon [26].

The spread of these mutations in wild populations of *An. gambiae* threatens the effectiveness of malaria vector control strategies based on the use of chemical insecticides and prompts surveillance and monitoring [27].

In this study, we determine the insecticide susceptibility status of *An. arabiensis* populations from Northern Sudan, find out if there is an association between susceptibility status and *kdr* mutations, and determine the effect of agricultural pesticide on susceptibility status of *An. arabiensis* within the study area.

## Methods

### The study area

The study was conducted in Northern Sudan state which is in desert and semi-desert areas. Two sites within the North Sudan state were chosen in this study divided into two categories: rural (Al-hamadab) and urban (Merowe).

### Al-hamadab

The Hamadab area is located between points E 18° 40.122′ N 32° 664.2′ and E 18° 40.336′ N 32° 800.4′ at Hamadab dam. In the northwest and points E 18° 35.318′ N 32° 208.1′ at the beginning of El dgaweet village at the east bank and E 18° 35.698′ N 32° 310.0′ at the beginning of El koaa’ village at the west bank in the southwest. The area is consisting of four-part Hamadab east at the west bank of the island, Hamadab west and east, Hamadab island, and the dam city. The length of the area is about 10 km and the average width is about 1.5 km. The main economic activity is agriculture.

### Merowe city

The area is in a semi-desert/desert area. Most of the residents live close to the Nile. The rainfall is rare and occasional. The maximum temperature reaches 47 °C from May to August and the minimum is about 7–10°C in winter. Agricultural activities depend on irrigation and influenced by the Nile flood and seasonal change in temperature. The cultivated land is extended along both sides of the Nile.

**Fig. 1:**
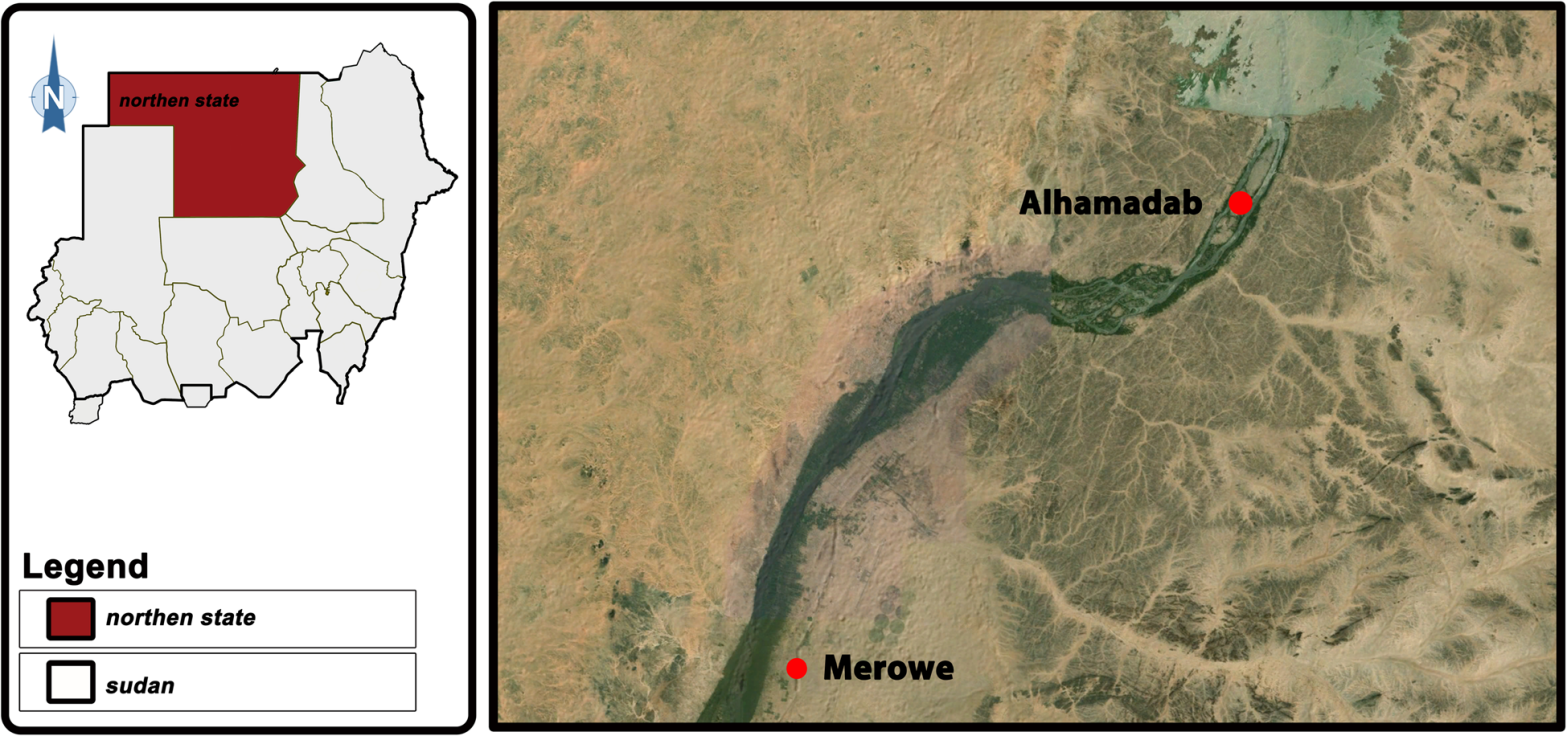
Map of the location of the study area in north Sudan state. Sudan map including urban site (Merowe) and rural site (Al-hamadab)

Merowe comprises a mixture of barren desert and urban sprawl that progressively becomes the dominant land use, while on the opposite bank the desert almost reaches the river (Fig. 1).

### Mosquito collections and rearing

*Anopheles sp.* larvae were scooped, using a dipper, from diverse habitats including fresh shallow pools of water, construction and sand winning sites, gutters, vegetable farming sites, and slow running streams.

Potential breeding sites of mosquitoes from the study area were collected 2–3 times per week. It is recommended that susceptibility tests are conducted using 1–3-day-old unfed female mosquitoes. In the field, this can only be obtained through the rearing of collected mosquito larvae [28].

Mosquitoes were reared at (25°±2°C) and at a relative humidity of 70–80%, with a 12:12 light to dark cycle and 45-min dusk/dawn period. Larvae were maintained in distilled water and fed on powdered yeast (Vital Brewer’s Yeast) and with about 100-mg fish meal every day. 10% of sugar solutions were provided for adults and were identified morphologically to *An. gambiae s.l*. using the keys of [2, 29]. Whenever enough was obtained (100 female mosquitoes per test, as recommended by the WHO), the resultant adult mosquitoes were used for insecticide tests.

### Insecticide susceptibility (bioassay test)

The choice of the insecticides, which were used in the study, was according to their chemical classification, their usage, the status of insecticide resistance in the area and to cover at least one insecticide in each class of insecticides.

Following WHO standard procedures [28], cohorts of non-fed adult females age 24–48 h post-emergence obtained from the larval collection were exposed to papers impregnated with WHO-recommended concentrations (V/W) of 0.75% permethrin, 4% DDT, malathion 5% and bendiocarb 0.1%. The control group was exposed to oil-treated control papers (without insecticide). Mosquitoes were exposed to the insecticide papers for 60 min, and during exposure time, the number of mosquitoes knocked down will be recorded on the susceptibility test form after 10, 15, 20, 30, 40, 50, and 60 min of exposure. Mortality was recorded after 24 h post-exposure.

The *An. arabiensis* colony F189 used in the present study was obtained from an insectary where it is under no insecticide selection pressure. It was stabilized in 2003 from Dongola, north Sudan. The F189 generation was exposed to insecticides from all four classes approved for use in malaria vector control: 5% malathion (organophosphate), 0.1% bendiocarb (carbamate), 4% DDT (organochlorine), and 0.75% permethrin (pyrethroids).

### Knowledge, Attitude and Practice (KAP) surveys of agricultural pesticide use

To obtain information on the use of pesticides among farmers, KAP surveys were carried out in the rural (Al-hamadab) and urban (Merowe) study sites. A sample of 62 farmers was recruited to answer the questionnaires.

### Detection of the *kdr* mutations

DNA extraction was done according to the method of Livak with some modifications [30]; single mosquitoes were homogenized in 1.5-ml Eppendorf tubes using a plastic pestle after adding 100 μl pre-heated (65 °C) Livak buffer; the homogenates were incubated at 65°C for 30 min. Potassium acetate was added to each tube and incubating the mixture on ice for 30 min. The mixer in each tube was centrifuged at 12,000*g* for 15 min at 4°C. Supernatants were transferred to clean tubes and 200μl ice-cold ethanol was added and centrifuged at 12,000*g* for 15 min at 4 °C. Pellets were rinsed in 100 μl 70% ice-cold ethanol, spun at 12,000*g* for 5 min at 4°C, and re-suspended in 50 μl in nuclease-free water. The knockdown resistance (*kdr*) genotypes were determined using two allele-specific PCR assays: (a) a diagnostic PCR developed by Martinez-Torres [19] was used for the detection of the leucine-phenylalanine *kdr* mutation in *An. gambiae* from West Africa and (b) Ranson [20] adapted this PCR for the detection of the leucine-serine *kdr* mutation in the Kenyan *An. gambiae* population. The two methods were used in this study for the detection of the West and East African *kdr* mutations in *An. arabiensis* permethrin /DDT susceptible and resistant specimens. Amplified fragments were analyzed in 1.5% agarose gel electrophoresis and visualized under UV light.

### Statistical analysis

Data were analyzed using a statistical package for social sciences SPSS version 20 for Windows (SPSS Inc, Chicago, IL, USA). The resistance status of mosquito samples was classified according to the WHO test procedures [28]. Consequently, the mortality rate of ≥ 98%, 90–97%, and <90% considered as susceptible, suspected/potential resistance, and resistant respectively. Fifty- and ninety-five percent knockdown times/minute (KDT50 and KDT95) were computed using probit analysis. Sampling sites and according to their landscape and economic activities were classified into urban (Merowe) and rural areas (Al-hamadab).

Generalized linear models (GLM) with a Poisson log-linear link function was run to examine the effect of sites, site types (urban and rural), and insecticides and their interaction on bioassay mortalities after 24 h. The test was also achieved for each insecticide independently with sites and site types as factors. A chi-square test was used to determine whether observed genotype frequencies are consistent with Hardy-Weinberg equilibrium. The association between the presence (yes/no) of *kdr* genotype and resistance phenotype (resistance/susceptible) was further confirmed for each insecticide using logistic regression.

## Results

### Bioassay results

**Fig. 2:**
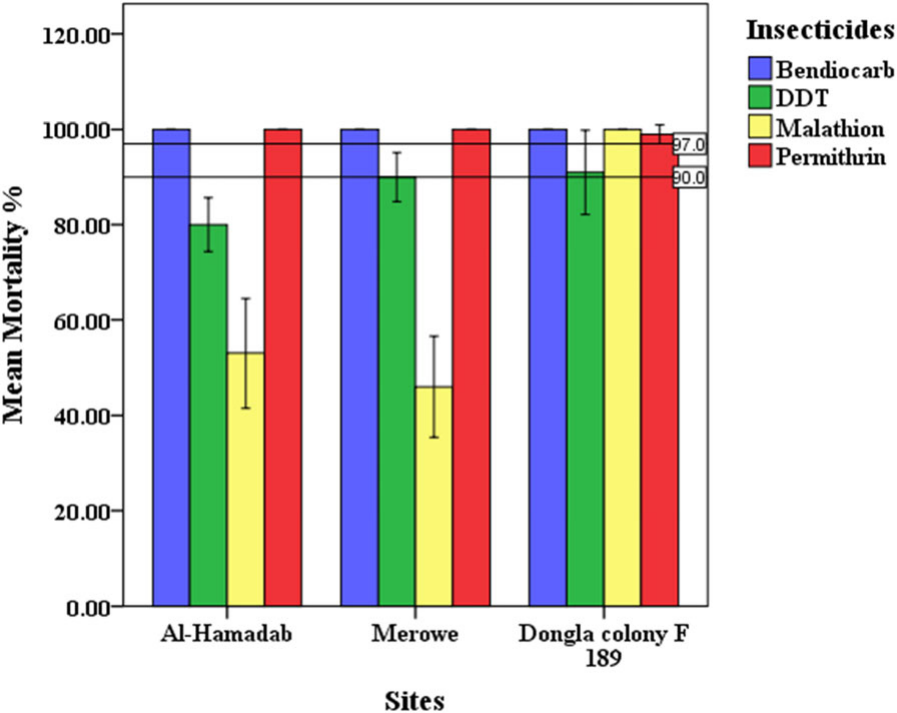
Mean mortality rates of *Anopheles arabiensis* exposure to bendiocarb, DDT, malathion, and permethrin. Mortality rates according to different sampling sites. Bars show mean mortality with standard deviation (SD). Solid horizontal lines show the WHO mortality threshold for the definition of resistant mosquito

**Fig. 3:**
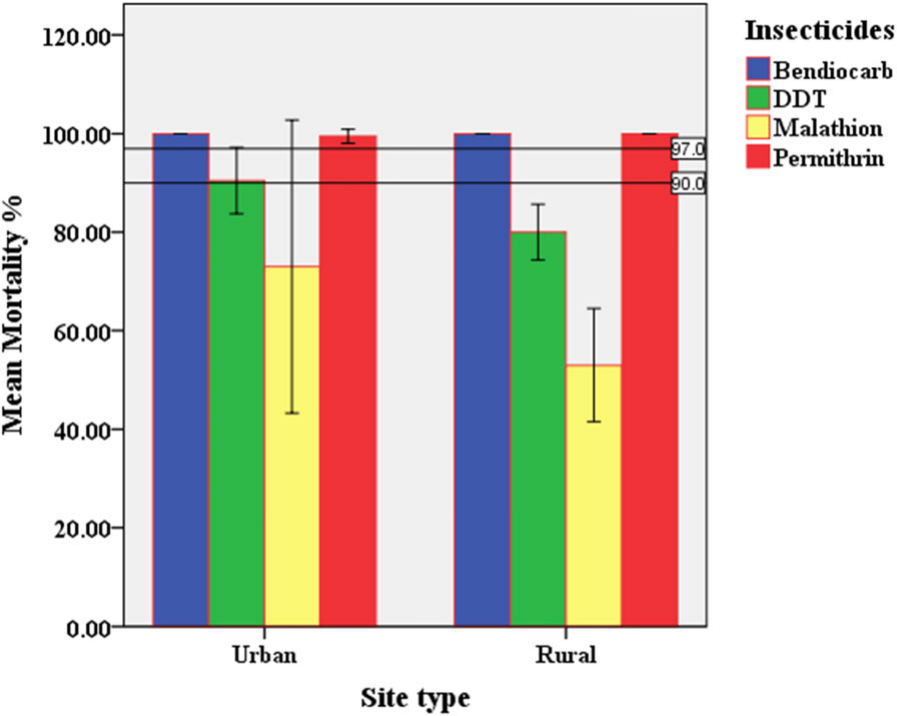
Mean mortality rates of *Anopheles arabiensis* exposure to insecticides. Mean mortality according to site type (urban and rural). Bars show mean mortality with standard deviation (SD). Solid horizontal lines show the WHO mortality threshold for the definition of resistant mosquitoes

The bioassay results after 24 h are illustrated in Table 1 and Figs. 2 and 3. According to the WHO criteria, all populations at the two sites would be considered as resistant to DDT and malathion. Permethrin and bendiocarb at the two sites showed 100% mortality rates; accordingly, their populations could be defined as fully susceptible, while the population of Dongola colony F189 showed mortality of 91% to DDT and hence would be considered as potentially resistant. Malathion susceptibility was found to be significantly low and showed 53% and 46% at Hamadab and Merowe respectively. In contrast, bendiocarb, malathion, and permethrin mortality showed susceptibility.

**Table 1:** WHO standard bioassay test on *Anopheles arabiensis*

Study area	Insecticides	Mortality (%) ± Std.D	Resistance status
**Al-Hamadab**	Bendiocarb (0.1%)	100 ± 00	**S**
	DDT (4%)	80.00±5.66	**R**
	Malathion (5%)	53.00±11.49	**R**
	Permethrin (0.75%)	100 ± 00	**S**
**Merowe**	Bendiocarb (0.1%)	100 ± 00	**S**
	DDT (4%)	90.00±5.16	**R**
	Malathion (5%)	46.00±10.58	**R**
	Permethrin (0.75%)	100 ± 00	**S**
**Dongola reference colony F189**	Bendiocarb (0.1%)	100 ± 00	**S**
	DDT (4%)	91.00±8.87	**PR**
	Malathion (5%)	100.00±0.00	**S**
	Permethrin (0.75%)	99.00±2.00	**S**

Mortality%: mortality rate 24 h after exposure to each insecticide. Number of tested mosquitoes per insecticide per site =100

*R* resistant, *PR* potential resistant, *S* susceptible

Generalized linear models (GLM) analysis indicated that insecticides, sites, site type, and their interaction were determinant factors on mortality rates (*P* < 0.01) (Table 2). Furthermore, the GLM test was applied for each insecticide independently. Sites and site types were considered as factors, while mortality rates as dependent variable (Table 3). Excluding malathion, mortality rates of all insecticides were not significant (*P* > 0.05) according to sites. Mortality rates of malathion and DDT were varied significantly (*P* < 0.0001 and *P* < 0.05 respectively) by site types, while mortality rates of bendiocarb and permethrin were not significant (*P* >0.05).

**Table 2:** Generalized linear model testing the effects of insecticide, site, and site type on bioassay mortality

Model factors included	Wald chi-square	df	Sig.
Insecticide	153.70	3	0.000
Site	16.06	1	0.000
Site type	10.53	1	0.001
Insecticide * site	114.86	3	0.000
Insecticide * site type	30.20	3	0.000

**Table 3:** GLM testing the effects of site and site type on bioassay mortality for each insecticide

Model factors included	Bendiocarb	DDT	Malathion	Permethrin
Sites	1	0.837	0.000	0.222
Site types	1	0.011	0.000	0.482

### Knockdown time thresholds

The 50% and 95% knockdown time thresholds (KDT50 and KDT95) calculated over 1 h against bendiocarb, DDT, malathion, and permethrin for each site are shown in Table 4. Overall, all populations from the two sites showed a faster knockdown time 50% to permethrin and bendiocarb than to DDT and malathion.

**Table 4:** Knockdown times (in minutes) (KDT50 and KDT95) of *Anopheles arabiensis* exposure to insecticides

Insecticides	Sites	KDT50 (95% confidence interval)	KDT95 (95% confidence interval)
Bendiocarb	Al-hamadab	15.91 (14.28–17.98)	31.15 (27.17–38.14)
	Merowe	32.38 (26.53–39.20)	58.80 (64.70–95.76)
	Dongola colony	16.64 (15.86–17.44)	26.88 (24.92–29.65)
DDT	Al-hamadab	41.23 (39.21–43.40)	79.19 (71.64–90.34)
	Merowe	38.44 (36.64–40.27)	69.22 (63.71–77.00)
	Dongola colony	47.06 (44.10–50.64)	91.29 (79.15–112.68)
Malathion	Al-hamadab	61.10 (55.29–69.95)	171.92 (133.22–251.78)
	Merowe	64.50 (59.65–72.79)	120.70 (99.05–169.30)
	Dongola colony	38.92 (37.07–40.71)	55.22 (51.68–60.60)
Permethrin	Al-hamadab	5.72 (3.09–7.40)	14.37 (12.64–17.54)
	Merowe	8.59 (7.16–9.65)	18.44 (16.54–21.77)
	Dongola colony	16.64 (15.86–17.44)	26.88 (24.92–29.65)

The rural area population (Al-Hamadab) was knocked down significantly faster with all examined insecticides because it had the lowest KDT50 except in DDT, wherein Dongola colony F189 population took significantly the longest time to be knocked down than the other population except in malathion.

### Knowledge, Attitude and Practice (KAP)

The KAP data shows that the most commonly cultivated crops are vegetables and beans in both winter and summer seasons. The practice of using pesticides was common among all farmers in different sites, with most sourcing the products from private suppliers. Out of six agricultural pesticides used, the focus was on the classes used by both farmers and public health authorities. Organophosphates were by far the most commonly applied pesticides, although there was no difference between urban and rural farmers in the pesticide classes used or the number applied. The farmers in both sites replaced the product with another class if efficacy was perceived to fall, but there was no significant difference in opinion as to whether poor pesticide application practice impacted efficacy. Farmers most commonly complained of mosquito bites at their homes during the summer season. Most farmers used mosquito nets for protection in the home. The summer considers the season of malaria peak in urban sites with winter as suggested by rural farmers (Table 5). Breeding sites observed in the urban sites were always road puddles, pools, and broken pipeline pools while in rural sites were mainly irrigation canals and hoof prints.

**Table 5:** Summary of KAP agro-sociological data: comparison of studied variables between urban and rural sites

Question	*χ*^2^	*P* value
Educational level	3.325	0.650
Kinds of summer crops	5.230	0.632
Kinds of winter crops	6.159	0.724
Use of pesticides	2.067	0.492
Source of pesticides used	3.286	0.193
Frequency of pesticide application	2.987	0.394
Do you think the poor use of pesticides causes loss of effectiveness?	2.162	0.339
Perceived seasonality of mosquito density	4.508	0.212
Is mosquito protection used?	3.685	0.815
Season of malaria appearance	21.099	0.000
Is mosquito protection used?	3.685	0.815
Season of malaria appearance	21.099	0.000

### *Kdr* allele frequency

Table 6 illustrates the existence of a 1014F-*kdr* allele in the three populations of *An. arabiensis* stratified according to whether they were resistant or susceptible after exposure to the WHO diagnostic dose of permethrin and DDT. The 1014S-*Kdr* allele was absent from all screened samples but the 1014F-*kdr* allele was detected. R allelic frequency was significantly higher in survivors for DDT (0.69 and 0.67). No RR genotype was identified among dead mosquitoes after DDT or pyrethroids exposure. In total, 141 samples were screened for the existence of a 1014F-*kdr* allele. The 1014F-*kdr* allele appeared in 107 specimens (75.88%) of which 96 (89.71%) were heterozygote. In summary, the resistant phenotype specimens constitute about 42% of heterozygote ones. The association between survivorship and the existence of a 1014F-*kdr* allele was measured by odds ratios and is illustrated in Table 6.

**Table 6:** L1014F alleles frequencies detected in mosquitoes of *An*. *Arabiensis* exposed to DDT and permethrin

Insecticides	Site	Phenotype	Genotype				Allele frequency				OR
			SS	RS	RR	Total	R	S	***χ***^**2**^	***P*** value	
DDT	**Dongola colony F 189**	**Susceptible**	4	8	0	12	0.33	0.67	3	0.08326	2
		**Resistance**	0	5	3	8	0.69	0.31	1.65	0.199	
	**Al-Hamadab**	**Susceptible**	3	17	0	20	0.43	0.57	10.93	0.000948	2.235
		**Resistance**	0	14	7	21	0.67	0.33	5.25	0.022	
	**Merowe**	**Susceptible**	8	2	0	10	0.1	0.9	0.123	0.725	6
		**Resistance**	0	10	0	10	0.5	0.05	10	0.0016	
Permethrin	**Dongola colony F 189**	**Susceptible**	17	2	0	19	0.05	0.05	0.059	0.809	1.5
		**Resistance**	0	0	1	1					
	**Al-Hamadab**	**Susceptible**	0	20	0	20	0.5	0.05	20	0.000008	-
	**Merowe**	**Susceptible**	2	18	0	20	0.45	0.55	13.39	0.00025	-

*R* resistant, *PR* potential resistant, *S* susceptible, *OR* odds ratio, *χ*^2^ chi-square value

## Discussion

This study showed that based on the WHO criteria for characterizing insecticide resistance/susceptibility, *An. arabiensis* from urban and rural localities (Merowe and Al-hamadab) were fully susceptible to bendiocarb and permethrin whereas resistant to DDT and highly resistant to malathion. The population of *An. arabiensis* from the reference Dongola colony F189 was susceptible to bendiocarb, malathion, and permethrin and potential resistance to DDT.

Intriguingly, despite the high level of compliance and long-term use of permethrin for public health purposes in Sudan [4, 7], the *An. arabiensis* mosquito population from North Sudan has not developed resistance to this chemical. A possible explanation is that its levels in agricultural use are below what would select for possible naturally occurring resistance in this species and the low-frequency use of pyrethroids in Northern Sudan state for public health.

The insecticide susceptibility data indicate high levels of resistance to malathion in *An. arabiensis* at all localities, the highest level of resistance as shown by the survival rates to malathion was recorded in Merowe (46%) followed by Al-hamadab (53%), and it is noteworthy that during the KAP agro-sociological surveys, it was noticed that malathion has been used in agriculture, indicating purchase from illegal markets. Although there was no significant difference in the type of pesticides used between urban and rural sites, farmers in the latter apply pesticides more frequently. Resistance to DDT and malathion is consistent with previously reported in *An. arabiensis* from El Rahad area (88% and 59% respectively) [16].

Knockdown time 50 was not significantly different between the rural and urban collections and the Dongola susceptible strain except in malathion. Besides, KDT50 and KDT95 for DDT and permethrin observed in the present study compare well with the previous study in Khartoum for *An. arabiensis* populations where mosquitoes are still relatively susceptible to DDT [31], whereas in the El Rahad area where specimens are highly resistant to DDT (67%), KDT50 is longer (more than the 67 min) [16].

The time to 50% knockdown for malathion at Merowe and Al-hamadab was higher (KT50 =64.50 min for Merowe and 61.10 min for Al-hamadab) than that of a susceptible laboratory colony (KT50=38.92).

The pyrethroid resistance levels in the study area at the present are unlikely to cause an epidemiological risk but regular periodic monitoring to establish levels of resistance is needed. *Anopheles arabiensis* from Merowe and Al-hamadab are susceptible to permethrin, and yet, their knockdown times are like those recorded in Khartoum 2007 [31]. In Gezira and Sennar, *An. arabiensis* showed high levels of resistance to permethrin (final mortalities varying between 10 and 55% and 6 and 22% respectively) [4]. Conversely, populations of this species proved susceptible to pyrethroids at three localities in the eastern part of the country. Recent study revealed that *An. arabiensis* remained susceptible to bendiocarb and permethrin in a border state with the Northern state [32]. This finding corroborates other studies from other parts of Sudan which also revealed [33].

The western *kdr* mutation, L1014F, was found in urban and rural sites whereas the eastern *kdr* mutation, L1014S, was not detected in any specimens. The absence of eastern *Kdr* mutation was confirmed by other studies in Khartoum and central Sudan [4, 7, 14].

The finding presented here for *An. arabiensis* showed no correlation between the resistant phenotype as ascertained by bioassay and the presence of the *kdr* mutation, with all individuals tested except the Merowe site which showed a moderate association with DDT (OR= 6).

Resistance to pyrethroids and DDT in *An. arabiensis* associates strongly with *kdr* [7, 21, 34]. However, a comparable correlation of *kdr* with the phenotypic expression of resistance could neither be established in a DDT-selected *An. arabiensis* laboratory strain from Sennar [35] nor in wild populations [4, 14].

The results suggest that the *kdr* genotype would be a poor indicator of phenotypic resistance to permethrin. Nevertheless, a moderate association between *kdr* and phenotype in DDT was proved which indicates the cross-resistance to permethrin could be much lower or absent. This finding is in concordance with [11].

The finding also suggests that the wild-type (Leu Leu) genotype among the dead individuals is more likely to associate with susceptible phenotype than resistance in the mosquito vector *An. arabiensis* from north Sudan.

## Conclusion

Overall, the results obtained in this study suggest good susceptibility of *An. arabiensis* in the study area to permethrin and bendiocarb (100% mortality); accordingly, they could be suitable insecticides for vector control activities, and the use of those insecticides for ITNs or IRS will be most likely promising.

The data also suggest that agricultural pesticides that are heavily used in both agriculture and public health might have a role in insecticide resistant of *Anopheles arabiensis*; the coordination between the agriculture and public health authorities regarding the use of those pesticides is recommended and developing rational management strategies for integrated insecticide-based control programs that can use both vectors and crop pests.

In general, the findings of *kdr* genotype emphasized a weak or moderate association between the presence of *kdr* and resistance phenotype for permethrin and DDT (*p* value is not significant in all of the sites), suggesting that *kdr* should not be used as the only indicator for monitoring insecticide resistance, other resistance mechanisms should be explored.

## Data Availability

All data generated or analyzed during this study are included in this published article.

## Abbreviations

*kdr*: Knockdown resistance
LLIN: Long-lasting insecticidal nets
IRS: Indoor residual spray
WHO: World Health Organization
GLM: Generalized linear models
OR: Odds ratio
KDT50: Knockdown time 50%
KDT95: Knockdown time 95%
KAP: Knowledge, Attitude and Practice
DNA: Deoxyribonucleic acid
DDT: Dichlorodiphenyltrichloroethane
PCR: Polymerase chain reaction
UV: Ultraviolet
SPSS: Statistical Packages for Social Sciences
